# Drug memory reconsolidation: from molecular mechanisms to the clinical context

**DOI:** 10.1038/s41398-023-02666-1

**Published:** 2023-12-01

**Authors:** Amy L. Milton

**Affiliations:** Department of Psychology, University of Cambridge, Cambridge, UK

**Keywords:** Learning and memory, Addiction

## Abstract

Since its rediscovery at the beginning of the 21^st^ Century, memory reconsolidation has been proposed to be a therapeutic target for reducing the impact of emotional memories that can go awry in mental health disorders such as drug addiction (substance use disorder, SUD). Addiction can be conceptualised as a disorder of learning and memory, in which both pavlovian and instrumental learning systems become hijacked into supporting drug-seeking and drug-taking behaviours. The past two decades of research have characterised the details of the molecular pathways supporting the reconsolidation of pavlovian cue-drug memories, with more recent work indicating that the reconsolidation of instrumental drug-seeking memories also relies upon similar mechanisms. This narrative review considers what is known about the mechanisms underlying the reconsolidation of pavlovian and instrumental memories associated with drug use, how these approaches have translated to experimental medicine studies, and the challenges and opportunities for the clinical use of reconsolidation-based therapies.

## Introduction

Drug addiction, or substance use disorder (SUD) is a chronic relapsing disorder that is estimated to annually cost the UK National Health Service in excess of £30 billion [1, 2], and to have an economic impact in the United States in excess of $440 billion per year [3]. Individuals addicted to drugs show high motivation for the drug(s) of abuse, impaired control over drug use, and persistent use of drug despite adverse physical, psychological and social consequences of drug use [4].

Addiction is a complex disorder, involving the hijacking of neural circuitry that has evolved to support motivated behaviour relevant to survival of the individual and propagation of the species. A prominent view is that learning mechanisms, originally evolved to support foraging behaviour, become maladaptively recruited to drug-seeking and drug-taking, allowing addiction to be conceptualised as a disorder of maladaptive learning and memory [5, 6]. This involves the aberrant engagement of both pavlovian and instrumental learning mechanisms. The acts of drug-seeking and drug-taking are instrumental, initially being goal-directed but, in a subset of individuals [7, 8], ultimately becoming habitual and compulsive [9, 10]. However, drug-seeking and drug-taking occur in specific environments (contexts) and in the presence of people and paraphernalia (i.e. discrete cues) that become associated with the drug high in a pavlovian manner [5]. These pavlovian drug-associated conditioned stimuli (‘CS-drug memories’) become powerful precipitators of relapse in those trying to maintain abstinence [11–24], and in experimental animal models of addiction, can be used to promote drug-seeking behaviour [25–29].

Conceptualising addiction as a disorder of learning and memory raises the prospect that drug-associated memories, whether pavlovian or instrumental, might provide targets for new treatment development [30–36]. A similar conceptualisation of other ‘maladaptive memory disorders’ such as specific phobia [37, 38] led to the development of treatments such as ‘cue exposure’ or ‘prolonged exposure’ therapy. Prolonged exposure therapy involves the repeated re-exposure of individuals to pavlovian CSs, previously associated with motivationally relevant outcomes, in the *absence* of that outcome [37]. For example, a phobic stimulus (e.g. a spider, normally eliciting fear in an individual with arachnophobia) is repeatedly presented while the individual works through guided exercises to control their fear (e.g. relaxation exercises). These repeated exposures can become progressively more proximal in a process referred to as ‘systematic desensitisation’ [39]. On successful completion of the therapy, the individual should no longer show the pavlovian conditioned response (i.e. fear) to the stimulus. However, despite its success for disorders such as phobia [40], the efficacy of prolonged exposure therapy for addiction is limited and mixed, with meta-analyses suggesting little to no effect on treatment outcomes [41–44].

Prolonged exposure therapy may be less effective in the treatment of addiction because rather than ‘erasing’ or ‘overwriting’ the original memory, the mechanism underlying prolonged exposure creates a new, inhibitory ‘cue-no outcome’ extinction memory that competes with the original for behavioural expression [45, 46]. While extinction learning is effective for disorders such as phobia [47], there are issues around its contextual specificity [46] (though see ref. [48] for methods to minimise the impact of contextual influences). However, the CSs associated with drugs of abuse often develop conditioned reinforcing properties that make them remarkably resistant to extinction [49, 50]. Conditioned reinforcement refers to the capacity of a pavlovian CS to acquire affective properties related to the primary reinforcer, and subsequently to support responding for the CS in its own right, allowing the cue to bridge long delays to primary reinforcement [51]. The fact that the CS becomes reinforcing in its own right is a major challenge to therapies based upon extinction learning.

Targeting the *consolidation* of memories underlying addiction – their initial storage within the brain, hypothesised to rely upon synaptic plasticity changes [52] and the formation of memory traces or ‘engrams’ [53, 54] – is not a feasible therapeutic strategy. Many who use drugs of abuse will ultimately not become addicted [55, 56] and thus, any globally-applied treatment strategy would target a large proportion of the drug-using population who will never develop SUD. Furthermore, even for those who would benefit from disrupting the consolidation of drug-associated memories, the window for treatment opportunity is very small, encompassing only a few hours (typically thought to be around 6 h, and certainly within 24 h - see ref. [57] for review). Although memory consolidation presents a challenging target for disrupting drug-associated memories, the rediscovery of memory *reconsolidation* at the beginning of the 21^st^ Century reignited interest in memory-based treatments for addiction.

**Fig. 1:**
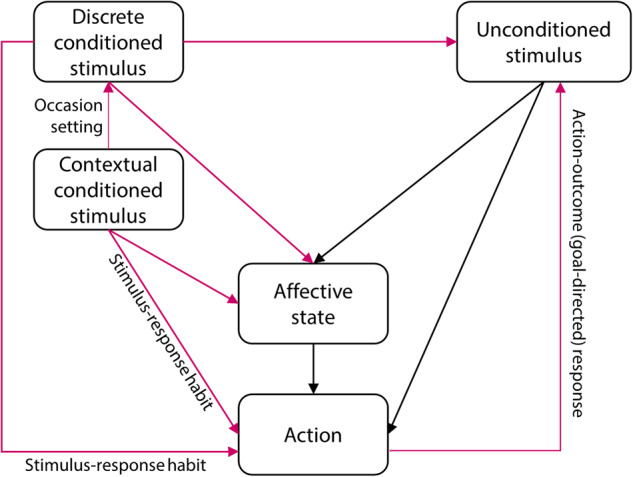
Simplified representation of associations acquired during learning about a drug of abuse. Associations are made between initially neutral stimuli (both discrete and contextual) that ultimately become associated with the drug of abuse, acting as an unconditioned stimulus, in a pavlovian manner. These associations can be affective or predictive, leading to actions (reflexive motor responses or more flexible instrumental behaviour). Additionally, instrumental associations are acquired between the action and the outcome (goal-directed responses) where individuals will work for the unconditioned stimulus if they are motivated to do so, and there is an instrumental contingency between the response and the unconditioned stimulus. Instrumental responding can also be supported by habitual, stimulus-response associations between conditioned stimuli and the action. Magenta lines represent learned associations.

Reconsolidation is hypothesised to be the process by which memories can be updated under certain conditions of retrieval, and almost since its rediscovery in 2000 [58] it has been proposed as a means by which old, well-established (and potentially maladaptive) memories could be targeted for disruption [31, 33, 59, 60]. By reactivating the maladaptive memories relevant to drug-seeking, it should be possible to make them once again susceptible to disruption through administration of amnestic agents, or behavioural interference. Reactivation (or, more mechanistically, destabilisation) of the memory can be achieved in different ways, but typically involves brief re-exposure to either the CS [61, 62] or the US [63, 64] for pavlovian memories (referred to as ‘CS-based’ and ‘US-based’ reactivation respectively), and a change in the reinforcement contingency for instrumental memories [65, 66]. Importantly, reactivation of the memory occurs under conditions in which there is a ‘mismatch’ between what is expected and what actually occurs [61], although the relationship between memory retrieval, memory destabilisation/reconsolidation and extinction learning is not straightforward [67] and likely interacts with prior learning history [68]. Thus, although reconsolidation-based approaches are simple in principle – induce lability of a well-established memory and administer an amnestic agent or behavioural interference procedure to disrupt its reconsolidation, thereby weakening it in the long-term - the practicalities of disrupting pavlovian and instrumental drug-associated memories has presented multiple challenges, including identification of the optimal associations to target for disruption (Fig. 1). In principle, these associations can be dissociated experimentally and could be independently targeted for disruption under the appropriate conditions of memory reactivation. In practice, these associations act simultaneously in the real world to support ongoing drug-seeking behaviour and to promote relapse during abstinence [31]. Therefore targeting multiple associations, whether simultaneously through reactivating by re-exposure to the drug US [63, 64], or sequentially by reactivating different CSs individually, may be the optimal approach for reconsolidation-based therapies.

The past two decades have seen a concerted research effort to determine whether reconsolidation-based approaches could be used to treat SUD, primarily in animal models, but also in a growing number of studies in humans. This review will focus upon treatments aiming to disrupt the reconsolidation of pavlovian memories (which should reduce the capacity of drug-associated CSs and contexts to precipitate relapse) and the disruption of instrumental memories (which should reduce drug-seeking behaviour directly) in both conditioned place preference (CPP) and self-administration models, and also consider how these proof-of-principle approaches have translated to clinical populations.

## The reconsolidation of pavlovian drug-associated memories

Drug-associated CSs are powerful precipitators of relapse in individuals trying to maintain abstinence [13–15, 17] with imaging studies showing that activation of the limbic corticostriatal circuitry in response to drug CSs is predictive of subsequent relapse for individuals addicted to alcohol [11, 12], nicotine [19–21], opiates [22] and psychostimulants [23, 24]. Disrupting the reconsolidation of drug-associated CSs therefore presents a potential treatment target for reducing the impact of these CSs on relapse behaviour in the long term.

**Fig. 2:**
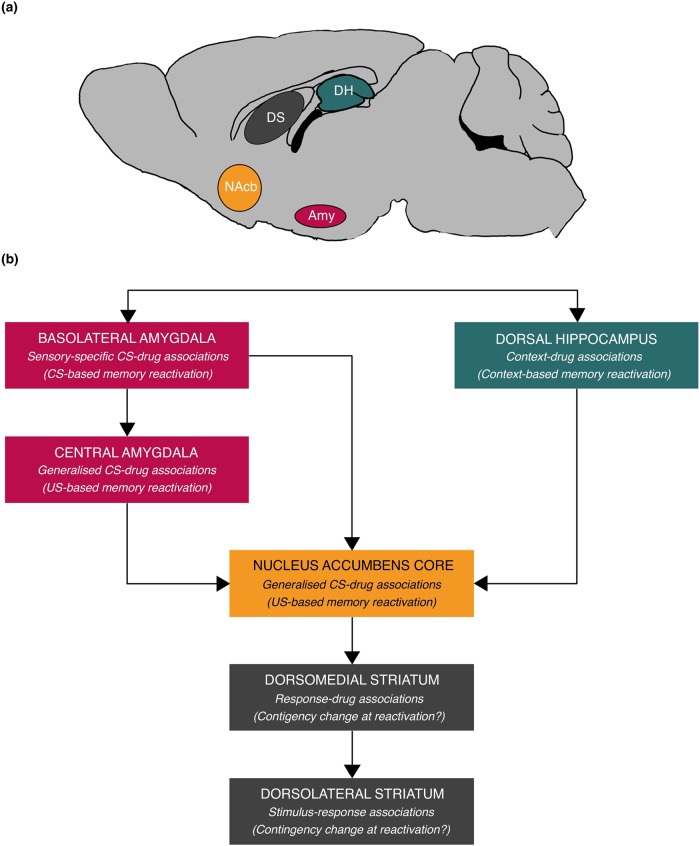
Neural regions implicated in drug memory reconsolidation. **a** Sagittal schematic of the rat brain, showing key regions of the corticolimbic striatal circuitry implicated in drug memory reconsolidation. **b** Connection of key brain regions implicated in drug memory reconsolidation, denoting associations supported by each structure and the type of memory reactivation session required to recruit it. Abbreviation: Amy amygdala, DH dorsal hippocampus, DS dorsal striatum, NAcb, nucleus accumbens. Reproduced, with permission, from ref. [69].

Numerous studies have investigated the impact of disrupting the reconsolidation of pavlovian drug-associated cues, in both self-administration and conditioned place preference (CPP; see below) procedures. Consistent with the activation of the limbic corticostriatal circuitry by drug-associated cues [13–15, 17], the reconsolidation of pavlovian CS-drug memories relies upon plasticity mechanisms within regions such as the basolateral amygdala (BLA), hippocampus and nucleus accumbens [69] (Fig. 2). The specific regions involved depend upon whether the memory being targeted for disruption is required for the association between contextual or discrete CSs and the drug outcome (unconditioned stimulus, US), and whether specific psychological processes (e.g. conditioned reinforcement) or the contribution of multiple psychological processes are being targeted. Indeed, even within regions, different behavioural procedures recruit specific ensembles that can be distinguished within the same animals using engram-labelling techniques [70]. However, for all these associations, the necessity of plasticity processes including activation of the NMDA subtype of glutamate receptor (NMDAR), protein kinase activation and protein synthesis has been demonstrated.

### Disrupting context-drug memories

Studies of the impact of contextual cues on drug-seeking, and whether reconsolidation of these memories could be a target for reducing that impact, have relied primarily upon the conditioned place preference (CPP) procedure, or have explicitly assessed the impact of context on the self-administration of drugs of abuse (e.g. refs. [71–74]). The CPP procedure involves training animals to associate a specific context with the effects of an experimenter-administered drug, and subsequently manifests as a preference for that context over an alternative context paired with a control injection [75]. By contrast, context-induced reinstatement involves training animals to self-administer drug (typically intravenously, but orally in the case of alcohol) in a specific context, and testing the capacity of that context to support recovery of drug-seeking compared to control contexts [76]. The memory representing the association of the context and the drug outcome is likely supported by the BLA [77–80], as it is for discrete cues [81–83]. However, the context representation itself depends upon the dorsal hippocampus, similar to the circuitry supporting contextual fear conditioning [84–86].

#### Disrupting the contextual memories underlying drug-conditioned place preference

**Fig. 3:**
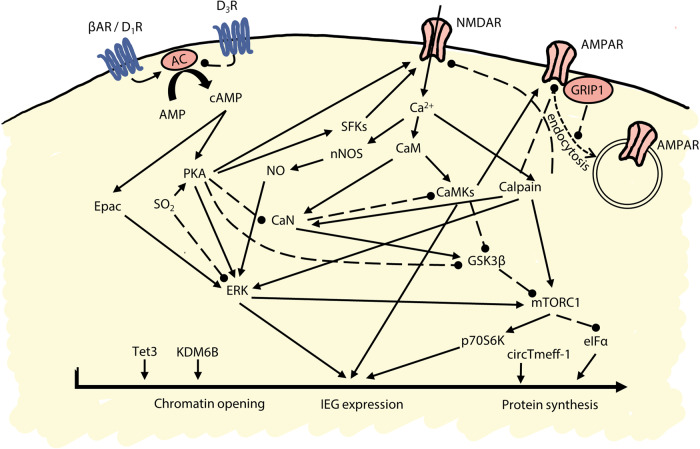
Crosstalk between molecular pathways modulating drug memory reconsolidation. Drug memory reconsolidation is modulated at the cell-surface, intracellular and nuclear levels, with crosstalk between the individual signalling molecules that have been investigated to date. Solid lines represent activation/enhancement of signalling; dashed lines represent inhibition. Abbreviations: AC adenylyl cyclase, AMP adenosine monophosphate, AMPAR α-amino-3-hydroxy-5-methyl-4-isoxazolepropionic acid subtype of glutamate receptor, βAR β-adrenergic receptor, cAMP cyclic adenosine monophosphate, CaM calmodulin, CaMKs calcium-calmodulin dependent kinases, CaN calcineurin, D_1_R D_1_ dopamine receptor, D_3_R D_3_ dopamine receptor, eIFα eukaryotic initiation factor α, Epac exchange protein activated by cAMP, ERK extracellular signal-regulated kinase, GRIP1 glutamate receptor interacting protein 1, GSK3β glycogen synthase kinase β, IEG immediate early gene, KDM6B lysine demethylase 6B, mTORC1 mammalian target of rapamycin complex 1, NMDAR N-methyl-D-aspartate subtype of glutamate receptor, nNOS neuronal nitric oxide synthase, NO nitric oxide, p70S6K p70-S6 kinase, PKA protein kinase A, SO_2_ sulphur dioxide, SFKs src-family kinases, Tet3 tet methylcytosine dioxygenase 3.

The memories underlying drug CPP can be disrupted by targeting biochemical pathways that ultimately lead to immediate early gene expression and protein synthesis (Fig. 3). These manipulations produce amnesia more reliably when the reactivation session is reinforced with an injection of the drug of abuse (i.e. ‘US-based’ reactivation is used). Protein synthesis inhibitors disrupt both cocaine [87] and morphine [87, 88] CPP when administered systemically in conjunction with reinforced re-exposure to the drug-paired context, as do local infusions of protein synthesis inhibitors intracerebroventricularly [89] or within the BLA [88] (though see ref. [90]), central amygdala [91], dorsal hippocampus [88] and nucleus accumbens core [88]. Upstream of protein synthesis, the reconsolidation of drug CPP memories depends upon the activation of transcriptional and translational regulators including eIFα [92], circTmeff-1 [93] and mTORC1 [94], and protein kinases including ERK [87], p70S6 kinase [94] and GSK3β [95, 96]. Protein phosphatases, such as protein phosphatase 1, are also necessary for the reconsolidation of cocaine CPP memories [97]. Furthermore, the knockdown of the immediate early gene *zif268* in the BLA or the nucleus accumbens disrupts the reconsolidation of the memories underlying cocaine CPP [98]. However, some proteases are also necessary for the reconsolidation of CPP memories, as the inhibition of the calcium-dependent cysteine protease calpain in the nucleus accumbens core prevents the reconsolidation of the memories underlying cocaine CPP by preventing its interaction with the scaffolding protein GRIP1 [99]. Epigenetic regulation is also critical for drug memory reconsolidation, as activation of the histone demethylase KDM6B in the medial prefrontal cortex [100] and Tet3 in the dorsal hippocampus [101] are both required for the reconsolidation of the memories underlying cocaine CPP.

At the level of cell surface receptors, catecholaminergic signalling appears necessary for the reconsolidation of the memories underlying drug CPP. Mice with a constitutive genetic knockout of the dopamine D_3_ receptor show impaired reconsolidation of cocaine CPP memories [102], as do wild type mice administered with the D_3_ receptor antagonist PG01037 [102] or the D_1_ receptor antagonist SCH23390 [103]. Furthermore, the enhancement of dopaminergic signalling with amphetamine facilitates the reconsolidation of the memories underlying morphine CPP [104]. Another catecholamine, noradrenaline, is also required for drug memory reconsolidation. Agonising α_2_ receptors with clonidine disrupts cocaine CPP [105], and antagonising β-adrenergic receptors with systemic propranolol during a single CPP memory reactivation session impairs subsequent CPP for cocaine [106], morphine [107] and nicotine [108], although not alcohol [109]. However, propranolol appears less effective when the drug CPP memory is strong, particularly if the memory has been recently acquired [110]. This boundary condition relating to memory strength may account for why some studies required multiple reactivation sessions under propranolol to observe subsequent amnesia [111]. Where propranolol is effective, it appears to exert its effects on memory via central noradrenergic signalling mechanisms. Administration of nadolol, a β-adrenergic receptor antagonist that does not cross the blood-brain barrier, does not disrupt the reconsolidation of the memories underlying morphine CPP under reactivation conditions for which propranolol is effective [107]. Furthermore, direct targeting of antagonists against α_1_ and β_2_ adrenergic receptors in the BLA disrupts the reconsolidation of the memories underlying cocaine CPP [112], as do both propranolol and nadolol when administered directly to the BLA [113]. β_1_ receptors in the central nucleus of the amygdala are also necessary for the reconsolidation of the memories underlying cocaine CPP [91]. Furthermore, administration of propranolol directly to the prelimbic cortex produces effects on both the retrieval and reconsolidation of cocaine CPP memories [113], perhaps indicating that some of the apparent discrepancies produced by studies using systemic propranolol can be attributed to effects in different neural structures.

The reconsolidation of the memories underlying drug CPP also depends upon activation at the NMDA subtype of glutamate receptor. Administration of the non-competitive NMDA receptor (NMDAR) antagonist MK-801 (dizocilpine) disrupts the reconsolidation of memories underlying cocaine CPP, usually with a single session of memory reactivation [114–116], but sometimes requiring multiple reactivation sessions [117]. Ketamine, another non-competitive NMDAR antagonist, disrupts the reconsolidation of memories underlying morphine CPP [118]. Amongst their many intracellular actions (Fig. 3), signalling at NMDARs leads to activation of neuronal nitric oxide synthase (nNOS) [119] and inhibition of nNOS also disrupts the reconsolidation of memories underlying cocaine CPP [115, 120]. Sulphur dioxide, which acts on nNOS pathways and affects neuronal function [121], impairs the reconsolidation of the memories underlying both cocaine and morphine CPP [122]. Finally, astrocytic lactate signalling, which likely exerts effects on synaptic plasticity via NMDARs [123], is also necessary for the reconsolidation of the memories underlying cocaine CPP [124, 125].

There has been great interest in whether behavioural interventions, aimed at exploiting the updating function of reconsolidation [126, 127], might reduce the strength of context-drug memories. Adapting the ‘retrieval-extinction’ procedure first used for updating fear memories by Monfils and colleagues [128] – in which a brief memory reactivation session, followed after a short delay by extinction training led to long-term reductions in fear memory – several studies have investigated whether reactivation of the drug-conditioned place preference memory can return it to a state in which it is susceptible to updating with the information that the context is no longer associated with drug. Xue and colleagues [129] reported reductions in both cocaine CPP and morphine CPP when CS-based reactivation of the CPP memory was followed by extinction training, though it has been reported that the initial reductions in place preference produced by the retrieval-extinction procedure can subsequently recover [130]. Differences in training history (e.g. the strength of conditioning) appear to alter the boundary conditions relevant to retrieval-extinction [131], allowing behaviour to be reduced either by a reconsolidation-dependent mechanism, or by facilitation of extinction. (Note that these two mechanisms are difficult to distinguish behaviourally [132]). Although the apparent discrepancies in the persistence of reduced preference for the drug-paired context following retrieval-extinction are unlikely due to the strength of conditioning (due to similar training protocols across studies), there may have been more subtle individual differences underlying the difference in treatment outcome [133]. An alternative updating approach, using counterconditioning (pairing a previously reward-associated CS to an aversive, rather than appetitive outcome) rather than extinction during the reconsolidation window, led to persistent reductions in both cocaine CPP [134] and alcohol CPP [135].

#### Disrupting the contextual memories influencing drug self-administration

In addition to supporting a preference for contexts paired with non-contingently administered drug, contextual cues also influence the propensity to seek drugs and to relapse following abstinence in individuals that have acquired drug self-administration [76]. Consistent with CPP studies, the reconsolidation of the memories underlying the influence of the drug-paired context on instrumental drug-seeking behaviour also relies upon protein synthesis [136], supported by intracellular signalling pathways activated by signalling at cell surface receptors (Fig. 3).

Consistent with its well-established role in representing contexts, much research into contextual influences on drug self-administration has focused upon the dorsal hippocampus. The projections between the dorsal hippocampus and basolateral amygdala are critical for the reconsolidation of memories underlying contextual influences on behaviour [72, 137], and disconnection of these structures during memory reactivation impairs subsequent context-induced reinstatement of drug-seeking [72]. Dorsal hippocampal activity is necessary for the reconsolidation of context-drug memories, as pharmacological inactivation with tetradotoxin [71] or optogenetic inhibition [138] of the dorsal hippocampus at reactivation impairs subsequent context-induced recovery of drug-seeking. However, inhibition of dorsal hippocampal protein synthesis with anisomycin at reactivation does not affect the capacity of the context to support the reinstatement of drug-seeking [71], although preventing signalling at GluN2A-containing NMDARs and activation of Src-family kinases does [139], suggesting engagement of some synaptic plasticity processes. As context-induced reinstatement of drug-seeking is subsequent impaired when anisomycin is delivered to the BLA [136] at reactivation, this has led to the hypothesis that the dorsal hippocampus modulates the reconsolidation of the context-drug memory, which may be stored within the BLA [138].

The plasticity mechanisms underlying the reconsolidation of the context memory within the BLA are like those described for the reconsolidation of the memories underlying CPP (Fig. 3). In addition to the requirement for protein synthesis [136], protein kinases including ERK [140] and PKA [141] (though not CaMKII [141]) are necessary. In contrast to studies of CPP, which have focused primarily upon monoaminergic and glutamatergic signalling at the cell surface level, studies of context-induced reinstatement have considered more extensively the requirement for glucocorticoid-mediated signalling and endocannabinoids. The glucocorticoid receptor antagonist, mifepristone, enhanced the reconsolidation of a context-cocaine memory [142] when administered directly to the BLA, similar to the effect of administering corticotropin-releasing factor (CRF) in female, but not male, rats [73]. CRF signalling in the BLA appears necessary for reconsolidation of the drug-context memory in both sexes, as administration of the CRF-1 receptor antagonist antalarmin at reactivation reduced subsequent context-induced cocaine-seeking in both males and females [73]. This activation of the HPA axis appears to interact with signalling at endocannabinoid receptors within the BLA. Systemic antagonism of CB1 receptors prior to, but not after, memory reactivation impairs the reconsolidation of the drug-context memory, and reduces the expression of immediate early genes including *zif268* in the BLA [143]. Furthermore, while direct antagonism of CB1 receptors within the BLA leaves drug-context memory reconsolidation unimpaired (suggesting that systemic administration of CB1 receptor antagonists exerts amnestic effects via structures other than the BLA), administration of the CB1 receptor agonist AM251 during memory reactivation facilitates subsequent context-induced cocaine-seeking [74], and increases plasma corticosterone levels in a manner comparable to return to the drug-associated context [74]. This may indicate that CB1 receptors modulate the degree of HPA axis activation during drug-associated memory reconsolidation.

A small number of studies have investigated the impact of behavioural interventions on the reconsolidation of drug-context memories. As for CPP [134, 135], aversive counterconditioning following a memory reactivation session can subsequently reduce context-induced alcohol-seeking [135]. Post-retrieval extinction produces a similar reduction in alcohol-seeking [144], although as a similar outcome is observed when extinction *precedes* memory reactivation, it is not clear that this effect depends upon memory reconsolidation rather than a facilitation of extinction learning [132, 133, 144].

#### Disrupting discrete CS-drug memories influencing drug self-administration

Discrete pavlovian CS-drug memories interact with the instrumental acts of drug-seeking and drug-taking in multiple psychologically and neurobiologically dissociable ways [31]. Three such processes that have been studied in the context of reconsolidation are conditioned reinforcement, conditioned approach, and conditioned motivation. Pavlovian conditioned approach can be observed in different individuals as ‘sign-tracking’ or ‘goal-tracking’, reflecting a tendency to approach the pavlovian CS or the location of US delivery when the CS is presented, respectively (see ref. [145] for review). Conditioned motivation is usually assessed through ‘pavlovian-instrumental transfer’ (PIT) procedures, and describes the capacity of reward-associated cues to influence instrumental behaviour associated with the same or different rewards (see refs. [146, 147] for review). These processes can be studied in isolation using specific behavioural procedures [148], though in reality drug-seeking will be influenced by all of these processes. This can be addressed experimentally with the use of reinstatement procedures [149], which have also been used to interrogate the mechanisms underlying the reconsolidation of pavlovian CS-drug memories.

The memories underlying the conditioned reinforcing properties of cocaine-associated cues, like those underlying CPP (Fig. 3), depend upon protein synthesis [62] and the expression of immediate early genes including *zif268* [62]. However, unlike CPP, the reconsolidation of the memory underlying conditioned reinforcement requires Zif268 expression in the BLA, but not the nucleus accumbens core [98]. Upstream of gene expression, the reconsolidation of the conditioned reinforcement memory depends upon PKA [150] and the activation of NMDA receptors [151] and β-adrenergic receptors (for both cocaine cues [152] and alcohol cues [153]), with the administration of the adrenergic prodrug dipivefrine enhancing reconsolidation of a CS-alcohol memory when given in conjunction with memory reactivation [153].

Less is known about the mechanisms underlying the reconsolidation of conditioned approach and conditioned motivation memories for drug-associated cues, which have been more extensively studied with natural (food) reinforcers [154–156]. However, it has been shown that the reconsolidation of pavlovian conditioned approach memories for CSs associated with alcohol [157] and sucrose [155, 156, 158–160] depend upon NMDAR activation, for both sign-tracking and goal-tracking responses. Although early studies suggested the memories underlying the goal-tracking responses were resistant to reconsolidation blockade [154], it has subsequently been shown that goal-tracking memories will reconsolidate under specific conditions [155, 158], and that the destabilisation of these memories relies upon dopaminergic signalling from the ventral tegmental area [159]. β-adrenergic receptor antagonism at reactivation has produced mixed results on the reconsolidation of conditioned approach memories, in contrast to its effects on the memories underlying conditioned reinforcement. Propranolol did not disrupt the reconsolidation of the memories underlying conditioned approach for either alcohol [157] or sucrose [156], and also did not disrupt the reconsolidation of memories underlying pavlovian-instrumental transfer under reactivation conditions in which NMDAR antagonism produced amnesia [156, 157]. Even when propranolol did disrupt the reconsolidation of a conditioned approach memory, only sign-tracking, and not goal-tracking, was impaired [161]. This has led to speculation that propranolol may impair the emotional component of the CS-US memory, without affecting the predictive value of CS [161]. This view would be consistent with studies of propranolol in fear-conditioned humans, in which automatic reflexive behaviours produced by an emotional CS were reduced by propranolol administration at reactivation, but the expectation that the CS would be associated with shock was unaffected [162].

Most studies assessing the mechanisms underlying the reconsolidation of CS-drug memories have used more translationally relevant procedures, in which the instrumental self-administration response for the drug produces both the drug reinforcer and the drug-associated CS, and the same response is tested following the manipulation aiming to disrupt reconsolidation [149, 163]. Reinstatement can subsequently be assessed through spontaneous recovery of previously acquired drug-seeking, or CS-induced or drug-induced reinstatement [163]. Like the memories underlying CPP, CS-drug memories acquired during self-administration depend upon protein synthesis [70, 164–166] and expression of the immediate early gene *zif268* in the basolateral amygdala [167]. Prevention of transcription by inhibiting the dephosphorylation of eIFα also disrupts CS-cocaine memory reconsolidation [92]. Epigenetic changes occur during the reconsolidation of CS-drug memories, as inhibition of DNA methyltransferase (DNMT) during reactivation impairs subsequently both CS-induced and drug-induced reinstatement for heroin [168] and cocaine [169]. Administration of garcinol, which reduces histone acetylation, impairs subsequent CS-induced reinstatement and the expression of the immediate early genes *arc* and *zif268* when administered into the lateral amygdala in conjunction with CS-cocaine memory reactivation [170]. Furthermore, garcinol can disrupt simultaneously the reconsolidation of multiple CS-cocaine memories when it is administered in conjunction with US-based reactivation [64].

Numerous protein kinases are required for the reconsolidation of CS-drug memories, including glycogen synthase kinase 3β (GSK3β), inhibition of which within the BLA, but not the CeN, impairs both subsequent CS-induced and drug-induced reinstatement [171]. Systemic administration of rapamycin, which inhibits mTOR signalling, similarly impairs the reconsolidation of CS-cocaine memories [172], and CaMKII inhibition not only impairs CS-cocaine memory reconsolidation, but also appears to facilitate its extinction [173]. Inhibition of PKA with Rp-cAMPS impairs CS-cocaine memory reconsolidation [150], as does activation of the exchange protein directed activated by cAMP (Epac) [174]. As for CPP, protein phosphatases are required for CS-drug memory reconsolidation, with inhibition of calpain in the nucleus accumbens core at reactivation reducing subsequent CS-induced cocaine-seeking [99]. Activation of the protein phosphatase calcineurin not only appears to disrupt the reconsolidation of CS-drug memory, but also appears to facilitate its extinction [175], similar to the effects of inhibiting CaMKII [173].

Monoaminergic signalling is also required for drug memory reconsolidation. Compared to CPP, dopaminergic signalling has been much less studied using self-administration procedures, but both D_1_ and D_3_-mediated signalling are necessary for the reconsolidation of CS-cocaine memories in mice [102, 176]. By contrast, noradrenergic signalling has been more extensively investigated, but has produced some apparently conflicting results in the literature that remain to be reconciled. Despite only being necessary for one of the three ‘routes to relapse’ [31] when CS-based reactivation procedures are used [152, 156, 157], signalling at β-adrenergic receptors is more effective in impairing CS-drug memory reconsolidation in reinstatement procedures when administered in conjunction with US-based reactivation. Propranolol administration in conjunction with a CS-based memory reactivation session gave mixed effects on the subsequent reinstatement of drug-seeking, producing no effect on the reinstatement of cocaine-seeking after enforced abstinence [177] or extinction of cocaine-seeking responses [164], but reducing subsequent alcohol-seeking after enforced abstinence [178] (with repeated rounds of propranolol administration and reactivation), and heroin-seeking in an extinction-reinstatement procedure [179]. Whether this reflects a difference in the noradrenergic mechanisms underlying the reconsolidation of memories associated with psychostimulants and depressants, or is related to the specific behavioural procedures used, warrants further investigation. However, when exposure to the drug US was used to reactivate the memory, β_1_ adrenergic receptor antagonism within the CeN impaired the reinstatement of cocaine-seeking after instrumental extinction [91], and administration of propranolol directly to the BLA reduced subsequent alcohol self-administration when alcohol reinforcers were available during the reactivation session [180]. The key adrenergic innervation supporting reconsolidation within the BLA appears to come from the nucleus of the solitary tract (NST) rather than the locus coeruleus (LC), as reactivation of CS-morphine memories led to increases in activation (measured through immediate early gene expression) in the NST and BLA, but not the LC [181]. Moreover, chemogenetic activation of NST-BLA projections (but not LC-BLA projections) led to greater sensitivity of the CS-drug memory to protein synthesis inhibition, while inhibition of these projections prevented the amnestic effect usually produced by protein synthesis inhibition [181]. However, these latter data are more consistent with noradrenaline from the NST supporting the destabilisation of the CS-drug memory, in contrast to the effects on restabilisation of the CS-drug memory reported above (see also refs. [113, 182]). They also stand in contrast to findings from the fear memory reconsolidation literature, where inhibiting noradrenergic signalling via LC-BLA projections *rescued* sensitivity to protein synthesis inhibition in a strong fear memory that was otherwise resistant [183]. Whether the source of noradrenaline input determines whether noradrenergic signalling influences memory destabilisation or restabilisation remains a question for future research.

Glutamatergic signalling is critical for cue-drug memory reconsolidation, and silent synapses within the nucleus accumbens appear to be unsilenced during drug memory reconsolidation, via a mechanism dependent upon the intracellular signalling molecule Rac1 [184]. Systemic antagonism of NMDARs at reactivation impairs cue-alcohol [166, 185, 186] and cue-cocaine [151] memory reconsolidation, as does antagonism of NMDARs within the BLA (which also reduces the expression of Zif268 in a reactivation-dependent manner) [151] and antagonism of GluN2A-containing NMDARs in the infralimbic cortex [187]. Systemic enhancement of NMDAR-mediated signalling with the partial agonist D-cycloserine enhances the reconsolidation of CS-cocaine memories, and increases Zif268 expression within the BLA [188]. Inhibition of lactate signalling at reactivation also reduces subsequent cocaine-seeking [125], potentially via effects on NMDARs.

As for CPP procedures, there has been interest in non-pharmacological interventions targeting cue-drug memory reconsolidation, including retrieval-extinction. As for CPP, the data have been mixed and may have been influenced by subtle differences in procedure (previous reviews have considered extensively procedural differences and their impact on retrieval-extinction outcomes for seeking behaviour for natural [189] and drug-associated [190] reinforcers) or individual differences in boundary conditions [133]. While the first demonstration of reduced recovery of drug-seeking following retrieval-extinction was effective for both opiate and cocaine memories [129], there have also been reports that a retrieval-extinction procedure that successfully impairs CS-nicotine memories is ineffective for CS-cocaine memories [191]. More research is needed to determine whether the retrieval-extinction procedure is supported by reconsolidation or extinction mechanisms [133].

## The Reconsolidation Of Instrumental Drug-Associated Memories

The acts of drug-seeking and drug-taking are supported by instrumental associations, and can be considered to be a form of aberrantly engaged, maladaptive foraging behaviour [5]. These associations are initially goal-directed (i.e. an association forms between the drug-seeking action and the drug outcome), but ultimately become habitual and elicited by environmental, pavlovian CSs (i.e. become supported by ‘stimulus-response’ associations) [10]. In a subpopulation of individuals, these drug-seeking habits ultimately become uncontrolled and compulsive in nature [9, 10]. Pavlovian and instrumental memories interact to support ongoing drug-seeking behaviour and to promote relapse during attempted abstinence (Fig. 1; see refs. [31, 32], for further detail) and many studies, including those discussed in the previous section, have measured reductions in the capacity of pavlovian CSs to influence instrumental responding following a disruption of pavlovian memory reconsolidation. Far fewer studies have attempted to disrupt the memories underlying the instrumental associations. However, disrupting the reconsolidation of the instrumental memories supporting drug-seeking and drug-taking behaviour would be an exciting therapeutic prospect.

It is likely that the relative paucity of studies of instrumental memory reconsolidation is at least partly due to an early study [192] in which an instrumental memory appeared not to undergo protein synthesis-dependent reconsolidation following retrieval. There may also be differences in the boundary conditions determining whether instrumental memories reconsolidate, as compared to pavlovian memories. Whereas pavlovian memories are susceptible to disruption following CS-based reactivation, instrumental memories may require a ‘mismatch’ in the type of reinforcement contingency experienced at reactivation [65, 66]. A predictable reinforcement schedule (i.e. additional sessions of training) did not render an instrumental memory underlying saccharin-seeking susceptible to disruption with cycloheximide [193] and studies investigating well-established cocaine-seeking memories in self-administering rats have indicated that a change in reinforcement schedule at reactivation, from a predictable ‘fixed-ratio’ schedule to a less predictable ‘variable ratio’ schedule of reinforcement, is required to induce susceptibility to disruption with the NMDAR antagonist MK-801, with non-reinforced memory reactivation sessions being insufficient to destabilise the instrumental memory [65, 66]. However, other studies of instrumental sucrose-seeking and nicotine-seeking have shown that non-reinforced memory reactivation sessions can lead to memory lability [194–196]. A detailed analysis of procedural differences that may have contributed to these apparent differences in boundary conditions is considered by Piva et al. [197], and may be related to the dose of amnestic agent administered, the potential engagement of metaplasticity mechanisms [194], the specific reinforcer, the strength of training, changes in reactivation context [198] or the passage of time between learning and reactivation [199]. It may be that a better understanding of the relationship between prior learning – which generates the expectations that determine whether a ‘mismatch’ or prediction error is detected [200] – and the reactivation session itself will be necessary to resolve these apparent differences [68].

If US-based reactivation session sessions are necessary to induce the destabilisation of instrumental memories, then this could present a challenge to clinical translation. Although prolonged exposure therapy often involves the handling of drug-associated paraphernalia [129] and, sometimes, limited self-administration of legal drugs such as nicotine (e.g. ref. [108]), a requirement to *self-administer* the drug of abuse would be challenging for ethical and safeguarding reasons in the case of illicit drugs. Whether non-contingent administration of the drug US (see Fig. 1), or use of pharmacological agents with similar neurochemical effects but reduced abuse liability (e.g. methylphenidate for psychostimulants [63] or methadone for opioids [201]), would be sufficient to destabilise the instrumental memory underlying drug-seeking remains an important outstanding question.

## Reconsolidation In The Clinical Context

To what extent can the findings discussed above, primarily from animal models relevant to drug addiction, be extrapolated to humans and to the clinical context? In light of the more extensive animal literature on disrupting the reconsolidation of pavlovian drug-associated memories, the majority of studies in humans have focused upon reducing reactivity to drug-associated CSs following reconsolidation-based manipulations. Many of these have recruited participants using legal drugs (alcohol and nicotine) but a small number of studies and clinical trials have focused upon psychostimulant and opiate users.

As for animals, pavlovian CS-drug memory reconsolidation depends upon NMDA receptor-mediated signalling in humans. The engagement of plasticity mechanisms appears to be important, as simply reducing neuronal activation with intravenous lidocaine infusions during reactivation had no effect on subsequent CS-induced cocaine craving [202].The administration of the NMDA receptor antagonist ketamine, in conjunction with a memory reactivation session that engaged prediction error, reduced subsequent alcohol-seeking in a population of hazardous drinkers [203], as did administration of the non-competitive NMDA receptor inhibitor nitrous oxide [204]. By contrast, another non-competitive NMDA receptor antagonist, memantine, given at memory reactivation was ineffective at reducing subsequent cigarette intake in a population of smokers [205]. However, despite both being NMDA receptor antagonists, memantine and ketamine have distinct effects on NMDA receptors [206] that may explain this apparent discrepancy.

Antagonism at β-adrenergic receptors has been used to target reconsolidation of addictive drug memories in both small-scale human trials and larger clinical trials. Despite initial reports that propranolol was ineffective at disrupting the reconsolidation of drug-associated memories in smokers when smoking-related CSs were used to reactivate the memory [207], subsequent studies using US-based reactivation of the nicotine memory showed a subsequent reduction in cigarette use in the group that had received propranolol [108]. The requirement for US-based reactivation to destabilise the memory may be specific to the strong interoceptive cues associated with smoking [208, 209], as reactivation of cocaine-associated memories through exposure to cocaine CSs was sufficient to induce sensitivity to disruption with propranolol in cocaine users [210, 211].

Numerous studies have investigated whether behavioural interventions might be used to interfere or alter reconsolidation of drug memories in humans. The types of interventions used include extinction training following memory reactivation (the ‘retrieval-extinction’ procedures discussed previously), counterconditioning, and cognitive reappraisal. The first demonstration of retrieval-extinction in drug users was that of Xue et al. [129], who built upon their rodent work to show in outpatient heroin users that a brief re-exposure to heroin-associated CSs, followed by prolonged exposure therapy, was sufficient to reduce CS-induced craving for at least 6 months [129]. A subsequent study showed that retrieval-extinction was also effective at reducing self-reported cigarette consumption in a population of smokers [212], although there were no effects on physiological reactivity to smoking-related CSs. This lack of physiological modulation was also found in a recent study [213], which also failed to replicate the reduction in cigarettes consumed in the retrieval-extinction group. However, they found that administering a stressful treatment (the Montreal Imaging Stress Test) prior to extinction itself led to reduced cigarette consumption. This is consistent with stress facilitating extinction learning, as has been observed previously for fear memories [214].

Several studies have investigated whether post-reactivation counterconditioning might be used to reduce subsequent drug use. In a population of hazardous drinkers, re-exposure to alcohol-associated CSs combined with visual counterconditioning (exposure to unpleasant images from the International Affective Picture Scale [215]) and gustatory counterconditioning (exposure to solutions laced with Bitrex) was sufficient to reduce alcohol consumption [216] for at least 9 months follow-up [217]. The effects of counterconditioning appeared stronger than those of cognitive reappraisal following memory reactivation, which reduced scores on an alcohol fluency task, but not subsequent alcohol consumption or attentional bias towards alcohol-associated CSs [218]. However, this intervention was brief compared to the procedures usually used in cognitive therapy, and likely did not reproduce the therapeutic relationship necessary for the effectiveness of cognitive therapy (see ref. [219] for further discussion). More intensive and extensive cognitive therapy with cocaine use disorder patients showed enhanced efficacy of cognitive therapy when combined with memory reactivation procedures [220, 221]. These findings suggest that the addition of a brief CS reminder session to already established therapies, such as prolonged exposure and cognitive therapy, could markedly enhance patient outcomes, though more research is needed to test this in larger scale clinical populations.

## Conclusions

The conceptualisation of addiction as a disorder of learning and memory, combined with the re-emergence of research into the mechanisms underlying memory reconsolidation, provides the potential for the development of novel therapeutic approaches for treating addiction. There are a multitude of maladaptive emotional memories that contribute to the persistence of drug-seeking and that promote relapse following a period of abstinence, including pavlovian (contextual and discrete CSs) and instrumental memories. While there are many reports that reactivating drug memories through presentation of the US (i.e. allowing the administration of a small dose of the drug of abuse) allows for widespread disruption within the drug-associated memory network, this approach would present the ethical challenge of administering an (often illegal) drug of abuse to a patient who is trying to maintain abstinence. Whether drugs with a similar pharmacological action could substitute for the drug of abuse in a ‘US-based’ reactivation session is worthy of further study.

The clinical trials of reconsolidation-based intervention for the treatment of addiction have been relatively small-scale, but overall there is cause for optimism. Future clinical studies will need to address a number of research questions, including the optimal reactivation procedure(s) to induce drug memory destabilisation (and ideally, this would include the development of methods to measure online and in real-time the induction of memory lability [68]), whether there are predictable individual differences in patients that could be used to tailor the reactivation procedure [222], and whether the effects seen to date generalise to larger patient populations. However, the encouraging data to date suggest that there is potential for reconsolidation-based interventions in providing a greater range of treatment options for patients.

Questions also remain within the preclinical literature, including whether behavioural interference approaches such as retrieval-extinction are acting via reconsolidation or extinction mechanisms [133] and whether reconsolidation-based interventions are effective in animal models of compulsive drug-seeking [7, 8]. Demonstrations that instrumental drug-seeking memories can be disrupted [65, 66, 223] raises the exciting prospect that it may be possible to return drug-seeking from being habitual to goal-directed, although whether individuals treated in this fashion would reacquire habitual and compulsive drug-seeking remains to be established. At the very least, it seems feasible that reconsolidation-based interventions would serve to reduce the pernicious effects of drug memories in eliciting automatic drug-seeking behaviours sufficiently to allow other, complementary therapies such as contingency management or cognitive behavioural therapy to have a greater chance of success.
